# Evaluating the Correlation Between Anteroposterior Diameter, Body Surface Area, and Height for Liver Transplant Donors and Recipients

**DOI:** 10.1097/TXD.0000000000001630

**Published:** 2024-05-16

**Authors:** Christopher J. Little, Scott W. Biggins, James D. Perkins, Catherine E. Kling

**Affiliations:** 1 Department of Surgery, University of Washington, Seattle, WA.; 2 Department of Surgery, Clinical and Bio-Analytics Transplant Laboratory (CBATL), University of Washington, Seattle, WA.; 3 Division of Gastroenterology, Hepatology and Nutrition, Department of Medicine, University of Pittsburgh, Pittsburgh, PA.; 4 Division of Transplant Surgery, Department of Surgery, University of Washington, Seattle, WA.

## Abstract

**Background.:**

Small stature and female sex correlate to decreased deceased donor liver transplant (DDLT) access and higher waitlist mortality. However, efforts are being made to improve access and equity of allocation under the new continuous distribution (CD) system. Liver anteroposterior diameter (APD) is a method used by many centers to determine size compatibility for DDLT but is not recorded systematically, so it cannot be used for allocation algorithms. We therefore seek to correlate body surface area (BSA) and height to APD in donors and recipients and compare waitlist outcomes by these factors to support their use in the CD system.

**Methods.:**

APD was measured from single-center DDLT recipients and donors with cross-sectional imaging. Linear, Pearson, and PhiK correlation coefficient were used to correlate BSA and height to APD. Competing risk analysis of waitlist outcomes was performed using United Network for Organ Sharing data.

**Results.:**

For 143 pairs, donor BSA correlated better with APD than height (PhiK = 0.63 versus 0.20). For recipient all comers, neither BSA nor height were good correlates of APD, except in recipients without ascites, where BSA correlated well (PhiK = 0.63) but height did not. However, among female recipients, BSA, but not height, strongly correlated to APD regardless of ascites status (PhiK = 0.80 without, PhiK = 0.70 with). Among male recipients, BSA correlated to APD only in those without ascites (PhiK = 0.74). In multivariable models, both BSA and height were predictive of waitlist outcomes, with higher values being associated with increased access, decreased delisting for death/clinical deterioration, and decreased living donor transplant (model concordance 0.748 and 0.747, respectively).

**Conclusions.:**

Taken together, BSA is a good surrogate for APD and can therefore be used in allocation decision making in the upcoming CD era to offset size and gender-based disparities among certain candidate populations.

Small liver transplant candidates are less likely to be transplanted on the waitlist than larger counterparts.^1,2^ Small-stature candidates have longer transplant times, higher waitlist death, and higher waitlist dropout.^1-3^ Importantly, even when positioned at the top of the waitlist, the transplant rate is lower for small-stature candidates.^4^ This is well known to every transplant surgeon who has struggled to find an appropriately sized organ for this population, the reasons for which are multifactorial. First, small-stature candidates have a lower Model for End-Stage Liver Disease (MELD) score because of the impact of muscle mass on creatinine, which leads to a lower ranking on the waitlist and, consequently, higher waitlist deaths.^5^ Given that female individuals represent the majority of small candidates, this disparity could be partially overcome with the introduction of MELD 3.0, which grants additional points to the female sex; however, the size-related disadvantage is expected to persist.^6^

The other contributing factor, and perhaps most impactful, is access to appropriately sized grafts. For MELD-matched candidates, small stature portends a lower likelihood of finding a suitable graft. Most studies on this topic have compared female to male individuals, showing that female sex is associated with a greater risk of waitlist mortality and lower rates of transplantation when adjusting for MELD, as well as longer waitlist time.^7,8^ However, there have been 2 recent studies that further described this disparity based on size and not sex—one using candidate height as the measure of small stature,^1^ and the other using body surface area (BSA).^2^ Both studies showed that shorter height and lower BSA are associated with increased time to transplant, increased death or delisting, and decreased deceased donor transplant, irrespective of sex.^1,2^

These studies are particularly relevant because the liver transplant community in the United States is reconsidering liver allocation under the new framework of continuous distribution (CD). With CD, the inclusion of attributes other than medical urgency (ie, MELD) is being discussed, and candidate biology—including small candidate size, specifically—is under discussion.^9^ Furthermore, the National Academies of Sciences, Engineering, and Medicine Report specifically recommends “including a modifier based on body size to overcome the demonstrated disparities observed for patients of small size.”^10^

The size discussions in CD are underway and preliminary proposals include directing donors with smaller grafts to small-stature candidates preferentially. However, despite both height and BSA being predictors of poor waitlist outcomes for small-stature candidates,^1,2^ calls have been made to incorporate more detailed donor and recipient information into allocation.^11^ The anteroposterior diameter (APD) of the liver is a metric that is used clinically at several centers, where donor and recipient values are routinely measured via pretransplant cross-sectional imaging to allow for appropriate size-matching between pairs.^12,13^ It is well studied in the pediatric population for left and left lateral segments^14,15^ and used to predict large-for-size syndrome in whole liver transplant.^16^ The APD of the right lobe of the liver corresponds to the largest AP dimension and can be measured using a well-described and reproducible methodology^17^; however, it is not yet systemically recorded.^12,13,16^ In this context, we hypothesized that APD correlates to standard donor and recipient size measurements, such as BSA and height, which could therefore be used as surrogates in allocation policy making. Hence, we seek to study the correlation between BSA and height to APD for both waitlist candidates and donors and directly compare waitlist outcomes by BSA and height.

## MATERIALS AND METHODS

### APD Measurement and Correlation

**FIGURE 1.**
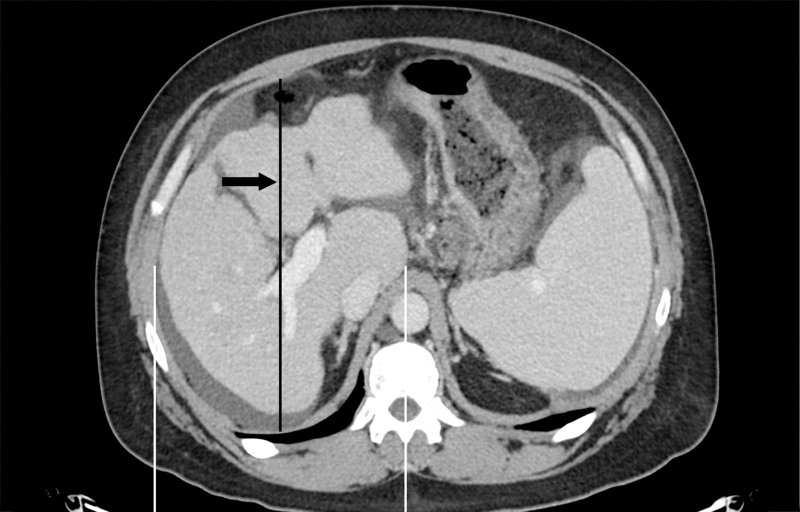
Sample APD measurement in a liver transplant recipient. The mid-hepatic point (black line and arrow) is identified halfway between the middle of the vertebral body and the inner abdominal wall (white lines) at the level of the right hepatic vein, and the APD of the space is then measured (length of the black line). APD, anteroposterior diameter.

Single-center deceased donor liver transplant (DDLT) recipients and their respective donors with available cross-section imaging were identified from calendar years 2021 to 2022. Donor–recipient pairs were excluded if either did not have cross-sectional imaging available. Height and weight were collected from the United Network for Organ Sharing (UNOS) Standard Transplant Analysis and Research file, and BSA was calculated using the Mosteller equation (√[height (cm) × weight (kg)] / 3600)^18^ based on the most recent height and weight recorded in the medical record before transplant. Similarly, APD was measured retrospectively from the most recent pretransplant imaging using the methodology described by Verma et al.^17^ Briefly, at the plane of the horizontal component of the main portal vein (PV), the halfway point was found between the mid-vertebral body and the right lateral margin of the abdominal wall, termed the mid-hepatic point. At this mid-hepatic point, the distance was measured between the anterior to posterior margin of the liver for donors or the anterior to posterior margin of the abdominal cavity for candidates (Figure 1). This difference was to account for the liver size of donors and the potential implantation space of candidates, given the impact of size expansion observed with ascites. Subanalysis was performed on the difference in APD between a recipient and their respective donor (APD difference = recipient APD minus donor APD). Cohorts were defined by APD difference >0 and APD difference ≤0. Categorical variables were reported in percentages and analyzed using the chi-squared test. Median values and interquartile ranges (IQRs) were reported for continuous variables, which were compared via Wilcoxon rank-sum tests.

For donors and candidates, we examined 3 correlation measures between APD, BSA, and height: Linear, Pearson, and PhiK correlation coefficients. PhiK is a new and practical correlation coefficient that works consistently between categorical, ordinal, and interval variables; captures nonlinear dependency; and reverts to the Pearson correlation coefficient in case of a bivariate normal input distribution.^19^ Knowing that the liver grows in 3 dimensions and not linearly, we hypothesized that the PhiK correlation may be better than a linear evaluation for correlation to APD.

### Compensation for Ascites

Methodology exists for quantifying ascites volume based on cross-sectional imaging, first described on the basis of patients with malignant ascites and validated in patients with cirrhotic ascites.^20,21^ As described, the thickness of ascites in centimeters was measured at 5 points—the bilateral subphrenic space, the bilateral paracolic space, and the pre-bladder space—and the average thickness was multiplied by the area of the standard abdominal cavity in the anterior projection (assumed to be 1000 cm^2^). When pelvic imaging was unavailable, we modified the formula to weigh the 4 upper abdominal measurements equally with the same assumption for the area of the abdominal cavity.

Operative notes were used to gather the volume of ascites present at transplant. For patients with ascites quantified at the time of transplant, a correlation was made between radiographic ascites and operative ascites. In these cases, the time between imaging and operation was recorded. Given the influence of ascites on weight and consequently BSA, we also stratified our correlation analysis by the presence of some ascites (either on imaging or at the time of transplant) versus no ascites (neither present on imaging nor at transplant). The threshold of >300 mL was used as the cutoff for the presence of ascites.

### Waitlist Outcomes

To expand the reach of this analysis to include waitlist candidates in addition to transplant recipients, UNOS data from June 18, 2013, to March 20, 2020, were used to perform a competing risk analysis of primary liver transplant waitlist candidates to determine whether height or BSA was a better predictor of waitlist outcomes. The multivariable competing risk model included prespecified risk factors, including female sex, diagnosis, hepatocellular carcinoma, age, race, blood type, ascites, PV thrombosis, previous abdominal surgery, MELD, life support, and diabetes. These variables were chosen on the basis of prior literature implicated in disparate waitlist outcomes.^22-29^ We examined the pairwise correlations among the independent variables to avoid multicollinearity in the multivariable competing risk model. Continuous variables with a correlation coefficient >0.80 were deemed highly correlated, suggesting potential collinearity issues. The point-biserial correlation coefficient measured associations between categorical variables and normal distributed continuous variables. Only one of the pairs of highly correlated variables was included in the model to prevent multicollinearity. The input for these analyses was obtained from the Organ Procurement and Transplantation Network (OPTN) data released April 1, 2021, through UNOS, which served as the contractor for the OPTN. The interpretation and reporting of these data are the responsibility of the authors and in no way should be seen as an official policy of, or interpretation by, the OPTN or the US Government. This study was approved by the University of Washington Institutional Review Board.

## RESULTS

### Anteroposterior Diameter

**FIGURE 2.**
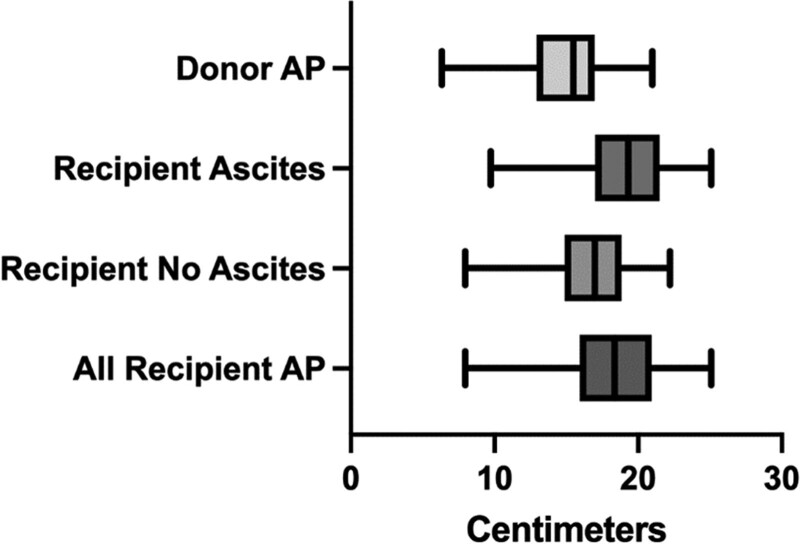
Graphic representation of median APDs among donors vs recipients. APD, anteroposterior diameter.

There were 143 donor–recipient pairs in the study. The median APD of the liver in donors was 15.5 cm (IQR, 12.95–17.0 cm), with a range of 6.35 to 21.0 cm. The median APD of the intra-abdominal space at the horizontal portion of the right PV in recipients was 18.35 cm (IQR, 15.94–20.93 cm), with a range of 7.96 to 25.09 cm. Among recipients without ascites, the median APD was 17.00 cm (IQR, 14.88–18.85 cm), whereas the median was 19.30 cm (IQR, 17.01–12.48 cm) in recipients with ascites. In this population of transplant pairs, donors trended toward having a lower APD than recipients (Figure 2).

**FIGURE 3.**
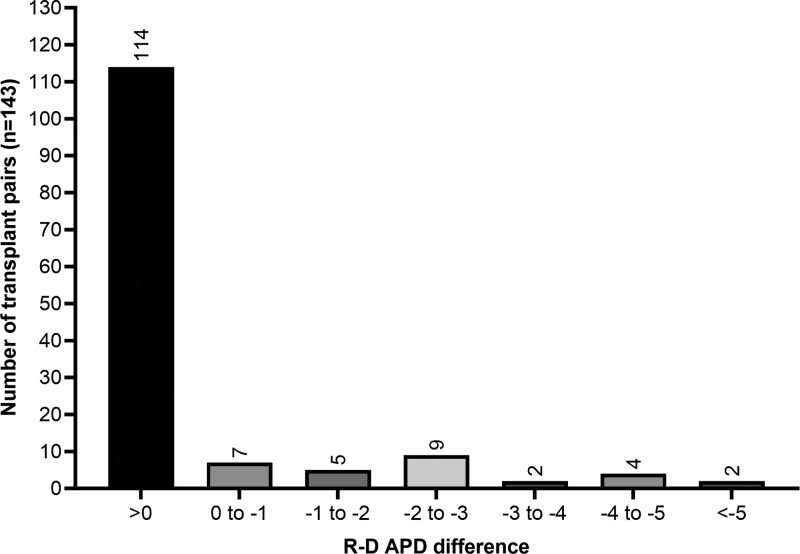
Donor–recipient pairs stratified by APD difference as measured by recipient APD minus donor APD (R-D APD). APD, anteroposterior diameter; R-D, recipient–donor.

In 20.3% of cases (n = 29), the APD of the donor liver exceeded the measured APD. In these 29 patients, 7 had an APD difference (recipient minus donor [R-D]) of 0 to –1 cm, 5 patients had –1 to –2 cm, 9 patients had –2 to –3 cm, 2 patients had –3 to –4 cm, 4 patients had –4 to –5 cm, and 2 patients had >–5 cm (Figure 3). In subgroup analysis, the median recipient APD was found to be larger for those in the APD R-D >0 group compared with the R-D ≤0 group (19.0 versus 14.7 cm, *P* < 0.001; Table 1). However, the median donor APD was smaller in the R-D >0 group than in the R-D ≤0 group (15.15 versus 16.5 cm, *P* < 0.001). Recipients without ascites comprised a higher proportion of the R-D ≤0 group (59%) than the R-D >0 group (30%, *P* = 0.004), and there was a trend toward the female sex in the R-D ≤0 group (45% versus 28%, *P* = 0.08). There was no difference in recipient height between groups (169 versus 175 cm, *P* = 0.14), although a trend toward lower BSA was observed in the R-D ≤0 group (1.95 versus 2.10, *P* = 0.051).

**TABLE 1.** Subanalysis of cohort characteristics between R-D APD >0 vs R-D APD ≤0

Characteristics	Overall (n = 143)	R-D APD >0 (n = 114)	R-D APD ≤0 (n = 29)	*P* ^*a*^
Donor				
Age	40 (29–52)	40 (29–54)	34 (28–46)	0.20
BSA	1.94 (1.78–2.13)	1.93 (1.78–2.11)	1.95 (1.77–2.23)	>0.9
Height, cm	173 (165–178)	173 (166–178)	173 (164–183)	0.70
Weight, kg	78 (67–94)	78 (67–92)	78 (67–95)	0.80
APD, cm	15.48 (12.95–16.95)	15.15 (12.42–16.79)	16.50 (15.48–17.74)	<0.001
Sex				0.50
Female	57 (40%)	47 (41%)	10 (34%)	
Male	86 (60%)	67 (59%)	19 (66%)	
Recipient				
Age	58 (48–64)	58 (48–63)	63 (55–65)	0.07
BSA	2.08 (1.82–2.26)	2.10 (1.91–2.28)	1.95 (1.76–2.17)	0.051
Height, cm	173 (165–181)	175 (168–180)	169 (160–182)	0.14
Weight, kg	88 (72–103)	91 (75–103)	80 (68–97)	0.048
APD, cm	18.4 (16.0–20.8)	19.0 (17.5–21.2)	14.7 (13.2–15.9)	<0.001
Sex				0.08
Female	45 (31%)	32 (28%)	13 (45%)	
Male	98 (69%)	82 (72%)	16 (55%)	
MELD	27 (20–31)	27 (20–31)	27 (16–31)	0.80
Ascites				0.004
None	51 (36%)	34 (30%)	17 (59%)	
Present	92 (64%)	80 (70%)	12 (41%)	

Data are presented as median (IQR) or n (%).

^*a*^Wilcoxon rank-sum test; Pearson’s chi-squared test.

APD, anteroposterior diameter; BSA, body surface area; IQR, interquartile range; MELD, Model for End-Stage Liver Disease; R-D, recipient–donor.

To better understand the size mismatch for transplants in which the donor APD measurement exceeded that of the recipient (R-D ≤0), we studied imaging for common anatomic variants. We found that 21 of 27 (72.4%) had high-riding kidneys (defined as the cephalad margin of the right kidney extending to or beyond the level of the main PV) requiring surgical mobilization during transplant, resulting in a smaller than expected measured candidate APD. Furthermore, among the 13 with APD R-D ≤–2.5, all had elevated renal anatomy. This contrasts the R-D >0 group, in whom no high-riding kidneys were identified on preoperative imaging. Of these 21 recipients, the majority (n = 15; 71.4%) also had right hepatic lobe atrophy, which occurred in the setting of preoperative transjugular intrahepatic portosystemic shunt (TIPS) procedure and right portal venous thrombosis in 7 and 5 recipients, respectively. Importantly, no recipients in this cohort had sequelae of large-for-size grafts, such as delayed abdominal closure, abdominal compartment syndrome, vascular complications, or primary nonfunction.

### Ascites Measurement

Of the 143 recipients, 74 (51.7%) had ascites on imaging, and of these, 69 (48.3%) had ascites at the time of transplant, whereas 5 (3.5%) no longer had ascites. Sixty-nine patients (48.3%) had no ascites on imaging, and of these, 18 (12.6%) had ascites present at the time of transplant, whereas 51 (35.7%) had no ascites at any time. The median time between imaging and transplant was 38 d (IQR, 15–79 d). Of the 74 patients with ascites on imaging, the median calculated volume was 1818 mL (IQR, 881–3349 mL), with a maximum of 6130 mL. The correlation between ascites on imaging and ascites at transplant was excellent for imaging done ≤14 d before transplant (*r* = 0.92, n = 23) and not as good for patients with imaging ≤30 d (*r* = 0.41, n = 42) or at any time (*r* = 0.61, n = 109) before transplant.

### Correlation Between BSA, Height, and APD

For donors, BSA correlated better with APD than height in all 3 measures of correlation (Table 2), with PhiK having the best correlation at 0.63. When assessing recipient all comers, neither BSA nor height correlated well with APD (all PhiK <0.5). Given the impact of ascites on BSA, we performed a subgroup analysis on recipients with no ascites (no ascites on imaging or at transplant) and those with ascites (ascites on imaging, at transplant, or both). For recipients with no ascites (n = 51), the levels of correlations were very similar to that of the donor group, with the BSA (PhiK = 0.63) out-performing height. In all recipients with ascites (n = 92), both correlations were poor.

**TABLE 2.** Correlation between APD of the liver, height, and BSA in donors and recipients

	Linear (R2)	Pearson	PhiK
Donors (n = 143)			
Height	0.03	0.19	0.20
BSA	0.24	0.50	0.63
All recipients (n = 143)			
Height	0.15	0.39	0.43
BSA	0.21	0.46	0.34
Recipients without ascites (n = 51)			
Height	0.18	0.44	0.24
BSA	0.31	0.57	0.63
Recipients with ascites (n = 92)			
Height	0.12	0.36	0.19
BSA	0.21	0.47	0.37

APD, anteroposterior diameter; BSA, body surface area.

An additional recipient subgroup analysis was performed to stratify recipients by sex (Table 3). Among female recipients, BSA correlated well to APD regardless of ascites status. Specifically, in female recipients without ascites (n = 17), BSA and APD were strongly correlated (PhiK = 0.80). In this same cohort, height and APD had a correlation of 0.60. Among female recipients with ascites (n = 28), APD correlated well to BSA (PhiK = 0.70) but was poorly correlated to height (PhiK = 0.38). APD correlated better to BSA (PhiK = 0.74) than height (PhiK = 0.63) in male recipients without ascites (n = 34) but had poor correlations to both (BSA PhiK = 0.38, height PhiK = 0.27) among those with ascites (n = 64).

**TABLE 3.** Correlation between APD of the liver, height, and BSA in recipients stratified by sex

	Linear (R2)	Pearson	PhiK
Female recipients without ascites (n = 17)			
Height	0.15	0.39	0.60
BSA	0.20	0.45	0.81
Male recipients without ascites (n = 34)			
Height	0.02	0.16	0.63
BSA	0.23	0.48	0.74
Female recipients with ascites (n = 28)			
Height	0.006	0.003	0.38
BSA	0.15	0.39	0.70
Male recipients with ascites (n = 64)			
Height	0.03	0.18	0.27
BSA	0.14	0.37	0.38

APD, anteroposterior diameter; BSA, body surface area.

### Waitlist Outcomes

Given that APD for donors or recipients is not currently measured or available in the OPTN data set, we sought to determine whether BSA or height predicts candidate waitlist outcomes. In the competing risk analysis for waitlist outcomes, there were 82 509 waitlist candidates with a cumulative incidence of 59.6% for deceased donor transplant, 10.5% for remaining on the waitlist, 16.9% for death or delisting, 3.0% for living donor transplant, 4.5% for improved clinical status, and 5.4% for other. Compared with remaining on the waitlist, in the univariable competing risk model, taller height and higher BSA were both associated with an increased hazard rate of DDLT, as well as decreased hazard rates of living donor liver transplant and clinical improvement (*P* < 0.001; Table 4). Univariable model concordance was slightly better for BSA (0.529) than for height (0.526).

**TABLE 4.** HRs (95% CIs) for univariable and multivariable competing risk analyses for liver transplant candidate waitlist outcomes with models including either height or BSA

	Remain on list	Died/too sick	DDLT	LDLT	Improved	Other	Model concordance
Univariable competing risk							
Height, m SHR, 95% CI	Ref	0.46 (0.39-0.54)	2.25 (2.06-2.46)	0.24 (0.17-0.36)	0.56 (0.41-0.77)	1.97 (1.49-2.61)	0.526
BSA, m^2^ SHR, 95% CI	Ref	0.93 (0.87-0.99)	1.44 (1.40-1.50)	0.28 (0.24-0.32)	0.54 (0.48-0.61)	0.99 (0.89-1.11)	0.529
Multivariable competing risk							
Height, m SHR, 95% CI	Ref	0.80 (0.67-0.96)	1.99 (1.78-2.20)	0.31 (0.21-0.46)	0.53 (0.36-0.78)	1.48 (1.09-2.01)	0.747
BSA, m^2^ SHR, 95% CI	Ref	0.82 (0.76-0.89)	1.13 (1.09-1.18)	0.32 (0.28-0.39)	0.80 (0.68-0.94)	0.87 (0.76-1.001)	0.748

BSA, body surface area; CI, confidence interval; DDLT, deceased donor liver transplant; LDLT, living donor liver transplant; SHR, subdistribution hazard ratio.

In the multivariable competing risk model for height, female sex was found to be collinear and was therefore excluded from the analysis. However, it was not collinear in the BSA model and, as such, was included. As in the multivariable analysis, taller height and higher BSA were significantly associated with increased access to deceased donor transplants, decreased delisting for death or clinical deterioration, and decreased living donor liver transplant (Table 4). The concordance of the height model was 0.747 and 0.748 for the BSA model.

## DISCUSSION

As the liver transplant community considers how best to overcome the disparate access to liver transplant for small-stature candidates, it is important to identify those who are underserved and allocate the livers that are appropriate for them. Liver APD is a clinically used measurement at many centers and is considered an important adjunct for size-matched allocation decisions.^12,13,16^ Indeed, at our transplant center, the majority of DDLT recipients in this population were transplanted livers from donors with appropriately smaller relative APDs. However, APD has not yet been used systemically nor is it recorded for donors or candidates in the UNOS database. For these reasons, we sought to explore the use of APD measurement in the coming era of CD by establishing its correlation to well-recorded size-related variables currently available in the national registry. This study shows that BSA correlates moderately well with donor APD, which is superior to its relationship with height. Likewise, the correlation between BSA and APD is strong for female recipients regardless of ascites status and male recipients without ascites. This contrasts with height, which was poorly correlated to APD in females with ascites, and was inferior to BSA among all populations. Importantly, both BSA and height are predictors of waitlist outcomes, including access to DDLT, with similar concordance in the multivariable models. These findings support the use of BSA as a surrogate for APD in size-related allocation policy and predictions of waitlist outcomes.

To our knowledge, this is the first study to examine the relationship between height, BSA, and APD based on radiographic measurements. Our findings are consistent with an autopsy study of 50 cadavers, in which the authors directly measured the APD of the liver and compared it with cadaver height and BSA.^30^ This showed a good correlation between APD of the liver and BSA (*R*^2^ = 0.643), and a poor correlation with height (*R*^2^ = 0.170), remarkably similar to our findings. A computed tomography–based volumetric study similarily demonstrated an association between right anteroposterior volume and greater “overall volume” or space for the liver in transplant recipients, as well as a greater cavitary volume in male recipients than female recipients despite similar rates of ascites.^31^ The authors also found that a formula using BSA best predicted available space for transplant. In addition, APD has also been used to predict large-for-size syndrome in adults and to guide the allowable thickness of liver segmental graft in pediatric recipients.^12,13,15,16^ Together, these studies support the use of APD for size-based allocation decision making.

Currently, it is not feasible to allocate livers based on APD, because it is neither consistently measured nor recorded for donors or recipients; hence, the establishment of a surrogate measure is important. BSA has been previously shown to correlate well with liver volume.^32^ Several papers have demonstrated a higher risk of graft failure when using livers outside a set donor-to-recipient BSA ratio (ie, donor BSA divided by recipient BSA), giving further validity to this measure and its use in identifying appropriately sized grafts for candidates.^33-35^ Additionally, Croome et al^36^ demonstrated that matching standardized total liver volume, which is calculated using BSA-based formula, has important prognostic implications, with donor–recipient ratios exceeding 1.25 portending higher risks of early allograft dysfunction and graft failure. In this study, we have shown that BSA correlates well to APD for all donors and recipients without ascites. Subanalysis additionally revealed that for all female recipients, a group that is well known to be marginalized on the liver transplant waitlist, a strong correlation between BSA and APD persists regardless of ascites status. Conversely, height does not correlate to APD among female recipients without ascites and is less correlated to APD than BSA among all other populations. Accordingly, Choukèr et al^37^ demonstrated that liver size is best predicted by age and body weight but not height. Furthermore, we are not aware of any such data that can be used to guide the matching of donors and recipients by height. Although the lack of correlation between APD and BSA in men with ascites is a notable shortcoming of this study, we speculate that the potential impact of inclusion of APD and BSA in the CD system could be influential in mitigating the disparities in waitlist outcomes observed among historically disadvantaged small-stature female candidates. Based on the collective body of literature on this subject as well as our data herein, if APD is not readily available, BSA is an effective surrogate, especially for small-stature female candidates and their potential donors.

The disparity among waitlist outcomes is multifactorial, although size and sex have been shown to strongly correlate with decreased access to allograft and, accordingly, higher waitlist mortality.^1,2,4,7^ In this context, the pertinence of this study lies in its attempt to augment liver allocation to mitigate the deleterious outcomes associated with these factors. Although APD and its surrogate BSA are measures of size, prior studies suggest that stature and liver size correlate with sex and, therefore, contribute, in part, to inequitable allocation to female candidates.^38,39^ Indeed, in our subanalysis, we found that recipients who received grafts from donors with larger relative APDs had smaller BSAs and were more likely to be female than those receiving livers from donors with smaller relative APDs. These findings suggest that small-stature female recipients are more likely to receive an oversized graft than their larger male counterparts within our study population. We therefore posit that in addition to the MELD 3.0 system, by considering APD and BSA in donor–recipient matching, the inequity experienced by female liver transplant candidates can be partially offset, thus improving access and overall outcomes. Although it is possible that the addition of these metrics may, in some cases, favor female candidates, they are intended to close the disparity faced by this population and should not lead to a disproportionate disadvantage for male recipients. Based on this study and others, we speculate that consideration of APD or BSA within the CD framework should improve allocation to small-stature candidates regardless of sex, thus enhancing equitable access to donor organs.^33-36^

In this retrospective review, we found that 20% of transplants in this era were performed using livers from donors with APDs greater than the recipient space. We posit that this disparity was influenced by high-riding renal anatomy within the recipient, which effectively reduced the intra-abdominal APD measured on preoperative imaging. This was particularly evident in those recipients on the extreme end of the APD R-D mismatch (≤–2.5), all of whom had elevated right kidneys. We think that this anatomic consideration was frequently influenced by factors that lead to atrophy of the right hepatic lobe, thus allowing for cephalad displacement of the right kidney. Specifically, a high percentage of recipients with elevated kidneys had previously received right PV to right hepatic vein TIPS procedures or had evidence of right PV thrombosis, both of which can result in loss of parenchymal volume. Although not described in granularity within the chart, the difference in APD observed in this setting was likely considered clinically insignificant at the time of allocation given the potential for right kidney mobilization during transplant, thus offsetting this size constraint. Alternatively, this cohort could reflect a change in ascites status between the time of imaging and transplant, which is an inherent constraint of the methodology. Importantly, none of the recipients within the APD R-D ≤0 cohort developed postoperative complications related to the large-for-size grafts. Although these findings demonstrate a limitation of the APD measurement, we think that this is strongly related to the retrospective review and does not subvert its clinical utility in allocation decision making. Furthermore, we acknowledge that the interpretation of these data may be skewed compared with national trends because APD is not universally used, although it is applied in donor–recipient matching at this center. This is to say that other programs may experience disproportionate numbers of R-D ≤0 cases based on alternative size-based metrics and allocation decision making, thus limiting the broad applicability of this subanalysis.

There were several additional limitations to our study. The BSA measure is impacted by weight, which can vary over time, particularly for patients with large volume ascites, introducing potential uncertainty to this measure. The interval between cross-sectional imaging and the date of transplant for some patients was quite long, leading to the possibility that ascites status on imaging, and hence APD measurement may not have been a true representation of the APD at the time of transplant. Specifically, progression and regression of ascites are nonlinear and variably affected by clinical decompensation or interventions such as paracenteses, diuretic management, and TIPS procedures, all of which can influence APD difference in both directions. However, 83.9% of patients had concordance between their imaging and operative ascites status. Furthermore, it is not common practice to obtain immediate preoperative imaging, suggesting that the interval time between imaging and surgery accurately represents clinical practice. Finally, as described previously, the retrospective design limits our ability to ascertain clinical and operative decision making as it relates to donor and recipient liver size based on the available imaging at the time.

As the liver transplant community moves toward CD, consideration should be given to the use of APD to size-match donors and recipients for DDLT. However, given that APD is not currently used or recorded systemically, we propose use of an appropriate surrogate for allocation purposes. The importance of BSA ratio matching is well described in the literature,^33-35^ which supports our findings that BSA correlates well to APD and has good predictive value for waitlist outcomes. We therefore posit that the inclusion of BSA into the allocation policy could serve to mitigate size and sex-related inequity and improve transplant access for small-stature candidates in the upcoming era of CD.

## ACKNOWLEDGMENTS

The authors thank Dr Guilherme Cunha for his assistance with obtaining an image for publication.

## References

[1] Bernards S Lee E Leung N. Awarding additional MELD points to the shortest waitlist candidates improves sex disparity in access to liver transplant in the United States. Am J Transplant. 2022;22:2912–2920. 35871752 10.1111/ajt.17159

[2] Kling CE Biggins SW Bambha KM. Association of body surface area with access to deceased donor liver transplant and novel allocation policies. JAMA Surg. 2023;158:610–616. 36988928 10.1001/jamasurg.2023.0191 PMC10061309

[3] Lai JC Terrault NA Vittinghoff E. Height contributes to the gender difference in wait-list mortality under the MELD-based liver allocation system. Am J Transplant. 2010;10:2658–2664. 21087414 10.1111/j.1600-6143.2010.03326.x PMC3059496

[4] Nephew LD Goldberg DS Lewis JD. Exception points and body size contribute to gender disparity in liver transplantation. Clin Gastroenterol Hepatol. 2017;15:1286–1293.e2. 28288834 10.1016/j.cgh.2017.02.033 PMC10423635

[5] Cholongitas E Marelli L Kerry A. Female liver transplant recipients with the same GFR as male recipients have lower MELD scores—a systematic bias. Am J Transplant. 2007;7:685–692. 17217437 10.1111/j.1600-6143.2007.01666.x

[6] Kim WR Mannalithara A Heimbach JK. MELD 3.0: the model for end-stage liver disease updated for the modern era. Gastroenterology. 2021;161:1887–1895.e4. 34481845 10.1053/j.gastro.2021.08.050 PMC8608337

[7] Locke JE Shelton BA Olthoff KM. Quantifying sex-based disparities in liver allocation. JAMA Surg. 2020;155:e201129. 32432699 10.1001/jamasurg.2020.1129 PMC7240642

[8] Cron DC Braun HJ Ascher NL. Sex-based disparities in access to liver transplantation for waitlisted patients with model for end-stage liver disease score of 40. Ann Surg. 2023;279:112–118. 37389573 10.1097/SLA.0000000000005933

[9] Organ Procurement and Transplantation Network. Request for feedback. Update on continuous distribution of livers and intestines. Available at https://optn.transplant.hrsa.gov/media/zc3lti1y/continuous-distribution-of-livers-and-intestines_liver_pc_winter-2023.pdf. Accessed February 1, 2023.

[10] National Academies of Sciences, Engineering, and Medicine. The National Academies Collection: reports funded by National Institutes of Health. In: Hackmann M English RA Kizer KW, eds. Realizing the Promise of Equity in the Organ Transplantation System. The National Academies Press; 2022. 35226429

[11] Hill AL Chapman WC. Addressing size-based disparities in liver transplant. JAMA Surg. 2023;158:617. 36988994 10.1001/jamasurg.2023.0195

[12] Jacob M Saif R Reddy J. Extreme large-for-size syndrome after adult liver transplantation: a model for predicting a potentially lethal complication. Liver Transpl. 2018;24:442–443. 29266684 10.1002/lt.24998

[13] Accardo C Vella I Pagano D. Donor-recipient matching in adult liver transplantation: current status and advances. Biosci Trends. 2023;17:203–210. 37344395 10.5582/bst.2023.01076

[14] Kanazawa H Sakamoto S Fukuda A. Living-donor liver transplantation with hyperreduced left lateral segment grafts: a single-center experience. Transplantation. 2013;95:750–754. 23503505 10.1097/TP.0b013e31827a93b4

[15] Namgoong JM Hwang S Song GW. Pediatric liver transplantation with hyperreduced left lateral segment graft. Ann Hepatobiliary Pancreat Surg. 2020;24:503–512. 33234754 10.14701/ahbps.2020.24.4.503 PMC7691208

[16] Allard MA Lopes F Frosio F. Extreme large-for-size syndrome after adult liver transplantation: a model for predicting a potentially lethal complication. Liver Transpl. 2017;23:1294–1304. 28779555 10.1002/lt.24835

[17] Verma SK McClure K Parker L. Simple linear measurements of the normal liver: interobserver agreement and correlation with hepatic volume on MRI. Clin Radiol. 2010;65:315–318. 20338399 10.1016/j.crad.2009.09.016

[18] Mosteller RD. Simplified calculation of body-surface area. N Engl J Med. 1987;317:1098. 3657876 10.1056/NEJM198710223171717

[19] Baak M Koopman R Snoek H. A new correlation coefficient between categorical, ordinal and interval variables with Pearson characteristics. Comput Stat Data Anal. 2020;152.

[20] Oriuchi N Nakajima T Mochiki E. A new, accurate and conventional five-point method for quantitative evaluation of ascites using plain computed tomography in cancer patients. Jpn J Clin Oncol. 2005;35:386–390. 15976067 10.1093/jjco/hyi109

[21] Wang R Qi X Guo X. Quantification of ascites based on abdomino-pelvic computed tomography scans for predicting the in-hospital mortality of liver cirrhosis. Exp Ther Med. 2017;14:5733–5742. 29285115 10.3892/etm.2017.5321 PMC5740797

[22] Haugen CE McAdams-DeMarco M Holscher CM. Multicenter study of age, frailty, and waitlist mortality among liver transplant candidates. Ann Surg. 2020;271:1132–1136. 30672803 10.1097/SLA.0000000000003207 PMC6639152

[23] Ziogas IA Hickman LA Matsuoka LK. Comparison of wait-list mortality between cholangiocarcinoma and hepatocellular carcinoma liver transplant candidates. Liver Transpl. 2020;26:1112–1120. 32475062 10.1002/lt.25807

[24] Kwong AJ Lai JC Dodge JL. Outcomes for liver transplant candidates listed with low model for end-stage liver disease score. Liver Transpl. 2015;21:1403–1409. 26289624 10.1002/lt.24307 PMC4838198

[25] Somsouk M Kornfield R Vittinghoff E. Moderate ascites identifies patients with low model for end-stage liver disease scores awaiting liver transplantation who have a high mortality risk. Liver Transpl. 2011;17:129–136. 21280185 10.1002/lt.22218 PMC3058247

[26] Leise MD Kim WR Kremers WK. A revised model for end-stage liver disease optimizes prediction of mortality among patients awaiting liver transplantation. Gastroenterology. 2011;140:1952–1960. 21334338 10.1053/j.gastro.2011.02.017 PMC4546828

[27] Montenovo M Rahnemai-Azar A Reyes J. Clinical impact and risk factors of portal vein thrombosis for patients on wait list for liver transplant. Exp Clin Transplant. 2018;16:166–171. 28621635 10.6002/ect.2016.0277

[28] Lai JC Ganger DR Volk ML. Association of frailty and sex with wait list mortality in liver transplant candidates in the multicenter functional assessment in liver transplantation (FrAILT) study. JAMA Surg. 2021;156:256–262. 33377947 10.1001/jamasurg.2020.5674 PMC7774043

[29] Kardashian AA Dodge JL Roberts J. Weighing the risks: morbid obesity and diabetes are associated with increased risk of death on the liver transplant waiting list. Liver Int. 2018;38:553–563. 28727287 10.1111/liv.13523

[30] Gupta M Sodhi L Yadav TD. Morphology of liver. Indian J Surg. 2008;70:3–7. 23133007 10.1007/s12262-008-0001-4 PMC3452600

[31] Addeo P Naegel B De Mathelin P. Predicting the available space for liver transplantation in cirrhotic patients: a computed tomography-based volumetric study. Hepatol Int. 2021;15:780–790. 33851323 10.1007/s12072-021-10187-6

[32] Urata K Kawasaki S Matsunami H. Calculation of child and adult standard liver volume for liver transplantation. Hepatology. 1995;21:1317–1321. 7737637

[33] Fukazawa K Yamada Y Nishida S. Determination of the safe range of graft size mismatch using body surface area index in deceased liver transplantation. Transpl Int. 2013;26:724–733. 23647566 10.1111/tri.12111

[34] Reyes J Perkins J Kling C. Size mismatch in deceased donor liver transplantation and its impact on graft survival. Clin Transplant. 2019;33:e13662. 31283049 10.1111/ctr.13662

[35] Kostakis ID Raptis DA Davidson BR. Donor-recipient body surface area mismatch and the outcome of liver transplantation in the UK. Prog Transplant. 2023;33:61–68. 36537056 10.1177/15269248221145035

[36] Croome KP Lee DD Saucedo-Crespo H. A novel objective method for deceased donor and recipient size matching in liver transplantation. Liver Transpl. 2015;21:1471–1477. 26358746 10.1002/lt.24333

[37] Choukèr A Martignoni A Dugas M. Estimation of liver size for liver transplantation: the impact of age and gender. Liver Transpl. 2004;10:678–685. 15108261 10.1002/lt.20113

[38] Mindikoglu AL Emre SH Magder LS. Impact of estimated liver volume and liver weight on gender disparity in liver transplantation. Liver Transpl. 2013;19:89–95. 23008117 10.1002/lt.23553 PMC3535518

[39] Darden M Parker G Anderson E. Persistent sex disparity in liver transplantation rates. Surgery. 2021;169:694–699. 32782116 10.1016/j.surg.2020.06.028

